# Expanded Newborn Screening for Inborn Errors of Metabolism and Genetic Characteristics in a Chinese Population

**DOI:** 10.3389/fgene.2018.00122

**Published:** 2018-04-20

**Authors:** Kejian Guo, Xuan Zhou, Xigui Chen, Yili Wu, Chuanxin Liu, Qingsheng Kong

**Affiliations:** ^1^Jining Maternal and Child Health Care Hospital, Jining, China; ^2^Department of Psychiatry, Jining Medical University, Jining, China; ^3^Shandong Key Laboratory of Behavioral Medicine, Jining Medical University, Jining, China; ^4^Collaborative Innovation Center for Birth Defect Research and Transformation of Shandong Province, Jining Medical University, Jining, China; ^5^Department of Biochemistry, Jining Medical University, Jining, China

**Keywords:** newborn screening, inborn errors of metabolism, incidence of IEMs, IEMs-associated gene mutation

## Abstract

The incidence of inborn errors of metabolisms (IEMs) varies dramatically in different countries and regions. Expanded newborn screening for IEMs by tandem mass spectrometry (MS/MS) is an efficient approach for early diagnosis and presymptomatic treatment to prevent severe permanent sequelae and death. To determine the characteristics of IEMs and IEMs-associated mutations in newborns in Jining area, China, 48,297 healthy neonates were recruited for expanded newborn screening by MS/MS. The incidence of IEMs was 1/1178 in Jining, while methylmalonic acidemia, phenylketonuria, and primary carnitine deficiency ranked the top 3 of all detected IEMs. Thirty mutations in nine IEMs-associated genes were identified in 28 confirmed cases. As 19 cases with the mutations in phenylalanine hydroxylase (*PAH*), solute carrier family 22 member 5 (*SLC22A5*), and methylmalonic aciduria (cobalamin deficiency) cblC type with homocystinuria (*MMACHC*) genes, respectively, it suggested that mutations in the *PAH*, *SLC22A5*, and *MMACHC* genes are the predominant causes of IEMs, leading to the high incidence of phenylketonuria, primary carnitine deficiency, and methylmalonic acidemia, respectively. Our work indicated that the overall incidence of IEMs is high and the mutations in *PAH*, *SLC22A5*, and *MMACHC* genes are the leading causes of IEMs in Jining area. Therefore, it is critical to increase the coverage of expanded newborn screening by MS/MS and prenatal genetic consulting in Jining area.

## Introduction

Inborn errors of metabolisms (IEMs) are a group of genetic diseases leading to severe complications in newborns, infants, children, adolescents, or adults. IEMs are caused by the defect of an enzyme, its cofactor or a transporter resulting in the accumulation of a substrate and/or the deficiency of its downstream products. Up to now, more than 500 IEMs have been detected. Although individual IEM is rare, the incidence of overall IEMs is high and varies dramatically in different countries and regions (Campeau et al., 2008). For example, the incidence of IEMs was reported to be 1/667 in Saudi Arabia (Moammar et al., 2010), 1/784 in United Kingdom (Sanderson et al., 2006), 1/2500 in Canada (Applegarth et al., 2000), 1/2900 in Germany (Lindner et al., 2011), 1/1944 in Egypt (Hassan et al., 2016), 1/2916 in Malaysia (Yunus et al., 2016), 1/2800 in South Korea (Yoon et al., 2005), and 1/3165 in Singapore (Lim et al., 2014). The incidence of IEMs is much lower in Japan, approximately 1/9000 (Yamaguchi, 2008; Yamaguchi et al., 2013).

Newborn screening for IEMs is an efficient approach for early diagnosis and presymptomatic treatment, which could improve the survival and well-being of affected infants. In addition to the classical newborn screening, expanded newborn screening by tandem mass spectrometry (MS/MS) has been applied widely since last decade. By analyzing amino acids and acylcarnitines in blood, three groups of IEMs could be simultaneously detected, including amino acid disorders, organic acid disorders, and fatty acid oxidation disorders. In China, expanded newborn screening started in 2004 and the incidence of IEMs was 1/3795 in a pilot study (Shi et al., 2012). However, later studies showed that the incidence of IEMs varied dramatically in different cities and regions of China, from 1/1683 to 1/8304 (Huang et al., 2011; Fan et al., 2013; Zhu et al., 2015; Lu et al., 2016). Expanded newborn screening for 25 IEMs is widely used in Jining since late 2014. To determine the incidence and characteristics of IEMs in Jining, 48,297 healthy neonates were recruited for expanded newborn screening of 25 IEMs by MS/MS. In the present study, we showed that the incidence of IEMs was 1/1178 in Jining, which is higher the incidence in China. In addition, 30 mutations in nine IEMs-associated genes were identified in the 28 confirmed cases and 19 cases with the mutations in phenylalanine hydroxylase (*PAH*), solute carrier family 22 member 5 (*SLC22A5*), and methylmalonic aciduria (cobalamin deficiency) cblC type with homocystinuria (*MMACHC*) genes, respectively, leading to the high incidence of phenylketonuria, primary carnitine deficiency, and methylmalonic acidemia, respectively. Our work indicated that the overall incidence of IEMs is high and the mutations in *PAH*, *SLC22A5*, and *MMACHC* genes are the leading causes of IEMs in Jining area. Therefore, it is critical to increase the coverage of expanded newborn screening by MS/MS and prenatal genetic consulting in Jining area.

## Materials and Methods

### Subjects

A total of 48,297 infants born in Jining, China, between January 2015 and December 2015, were recruited for expanded newborn screening by MS/MS. In all, 15,731 infants were born in the urban area and 32,566 infants were born in the suburban and rural areas. Informed and written consent was obtained from the parents. This study was approved by the Ethical Committee of Jining Medical University.

### Procedures of Screening Test and Follow-Up Testing

All the procedures were carried out in accordance with the Jining Newborn Screening Programme (2015) and Technical Guide of Newborn Screening in China (2010). Briefly, blood taken from heelstick was spotted on Whatman 903 filter paper, which was dried and sent by mail to the screening laboratory. The time of sampling was between the 3rd to 10th day of life. Dried blood spots were pre-processed following the instruction of NeoBase^TM^ non-derivatized MS/MS kit (PerkinElmer, MA, United States), and then they were analyzed by using TQD tandem mass spectrometry system (Waters, MA, United States) and NeoBase non-derivatized MS/MS kit (PerkinElmer, United States). The analytes include alanine (ALA), arginine (ARG), citrulline (CIT), glycine (GLY), leucine (LEU), methionine (MET), ornithine (ORN), phenylalanine (PHE), tyrosine (TYR), valine (VAL), free carnitine (C0), acylcarnitines (C2, C3, C3DC, C4, C4DC, C4OH, C5, C5:1, C5DC, C5OH, C6, C8, C8:1, C10, C10:1, C10:2, C12, C12:1, C14, C14:1, C14:2, C14OH, C16, C16:1, C16OH, C16:1 OH, C18, C18:1, C18:2, C18OH, C18:1OH) and succinylacetone. The normal ranges and cut-off values of analytes in the manual of NeoBase^TM^ non-derivatized MS/MS kit (Cat. 3040-001Z, PerkinElmer, MA, United States) and from the worldwide collaborative project were applied (McHugh et al., 2011). Suspected positive cases were recalled for the repeated test by MS/MS. The follow-up testing commences for the second time positive cases, including biochemical tests or genetic analysis. The recall and follow-up protocol in the guidelines “Follow-Up Testing for Metabolic Disease Identified by Expanded Newborn Screening Using Tandem Mass Spectrometry” was applied in our study (Bennett, 2009; Dietzen et al., 2009). Definitive diagnosis is made by specialists based on the clinical symptoms, screening test, and biochemical and genetic analysis. The parents of all cases with definitive diagnosis were informed and referred to specialists for the treatment.

### Screened Panel of IEMs

Twenty-five IEMs were screened in this study. Nomenclature and abbreviations are adapted from the guidelines “Follow-Up Testing for Metabolic Disease Identified by Expanded Newborn Screening Using Tandem Mass Spectrometry” (Bennett, 2009). The screened IEMs included hypermethioninemia, phenylketonuria, homocystinuria, maple syrup urine disease, tyrosinemia, citrullinemia, argininemia, very long-chain acyl-CoA dehydrogenase deficiency, long-chain 3-hydroxyacyl-CoA dehydrogenase deficiency, medium-chain acyl-CoA dehydrogenase deficiency, short-chain acyl-CoA dehydrogenase deficiency, glutaric acidemia type II, primary carnitine deficiency, carnitine palmitoyltransferase I (CPT I) deficiency, carnitine palmitoyltransferase II (CPT II) deficiency, trifunctional protein deficiency, multiple carboxylase deficiency, methylmalonic acidemia, glutaric acidemia type I, isovaleric acidemia, 3-hydroxy-3-methylglutaric aciduria, propionic acidemia, malonic aciduria, 3-methylcrotonyl-CoA carboxylase deficiency, and beta-ketothiolase deficiency.

### Genetic Analysis

Genetic analysis was performed by BioSan Biochemical Technologies (Hangzhou, Zhejiang, China) and Be Creative Lab (Beijing, China) using the Sequenom MassARRAY iPLEX platform (Sequenom, San Diego, CA, United States), respectively. Briefly, genomic DNA was extracted from whole blood using QIAamp DNA Micro Kit (Qiagen, Hilden, Germany). Primers were designed by using Typer 4.0 software (Sequenom, San Diego, CA, United States). A total of 942 sites in IEMs-associated genes, *ACADL*, *ACADM*, acyl-CoA dehydrogenase, C-2 to C-3 short chain (*ACADS*), *ACADVL*, *ACAT1*, *BTD*, *CPT2*, *ETFA*, electron transfer flavoprotein dehydrogenase (*ETFDH*), glutaryl-CoA dehydrogenase (*GCDH*), *HLCS*, *HMGCL*, *IVD*, *MCCC1*, *MCCC2*, *MMACHC*, methylmalonyl-CoA mutase (*MUT*), *PCCA*, *PCCB*, *SLC22A5*, *SLC25A20*, *ARG1*, *ASS1*, *BCKDHA*, *BCKDHB*, *CBS*, *DBT*, *FAH*, *MAT1A*, *MTHFR*, *OTC*, *PAH*, 6-pyruvoyltetrahydropterin synthase (*PTS*), *SLC25A13*, are included based on the mutations in PubMed, OMIM, and HGMD databases. The protocol of Sequenom iPLEX genotyping was strictly followed. One picomole PCR primers, 5 ng genomic DNA, and reaction mix (Sequenom, San Diego, CA, United States) were applied in each PCR. After SAP enzyme treatment and single-base extension, the products were spotted onto a SpectroCHIP with the Sequenom MassARRAY RS1000 Nanodispenser (Sequenom, San Diego, CA, United States). Mass determination was done with the Sequenom MassARRAY mass spectrometer (Sequenom, San Diego, CA, United States).

### Statistical Analysis

The data was presented descriptively and Chi-square tests were performed to compare the incidences or rates in the urban and suburban/rural areas. Statistical significance is accepted when *P* < 0.05.

## Results

### Incidence and Characteristics of IEMs

To investigate the incidence of IEMs in Jining, 48,297 infants were recruited for expanded newborn screening by MS/MS in Jining. Of 769, 725 positive cases were successfully recalled for the second examination by MS/MS. The follow-up testing immediately commences for the positive cases in the second MS/MS test, including plasma amino acid analysis, urine amino acid analysis, urine pterin metabolite analysis, enzyme activity analysis, and/or genetic analysis (Bennett, 2009; Dietzen et al., 2009). The parents of all cases with definitive diagnosis were informed and referred to specialists for the treatment. Forty-one cases with IEMs were confirmed by follow-up testing and clinical investigation. The incidence of IEMs was 1/1178 and the corrected incidence was 1/1110 (**Table 1**).

**Table 1:** Incidence and characteristics of IEMs.

	Urban	Suburban and rural	χ^2^	*P*-value	Total
Recruited	15,731	32,566			48,297
Presumptive positive (positive in first screening)	255	514			769
Positive rate (%)	1.62	1.58			1.59
Recalled	249	476			725
Recall rate (%)	1.58	1.46	8.03	0.005	1.50
Definitive diagnosis	6	35			41
Positive predictive value (%)	2.41	7.35	7.49	0.006	5.66
Incidence (1 in)	2622	930	6.01	0.014	1178
Corrected incidence (1 in)	2560	862			1110

A total of 15,731 neonates in the urban area and 32,566 neonates in the suburban or rural area were screened. Six and 35 confirmed cases were detected in the urban area and the suburban or rural area, respectively. The incidence of IEMs in the suburban or rural area was significantly higher than that in the urban area, 1/930 vs. 1/2622 (*P* < 0.05; **Table 1**). Based on the positive predictive values, 2.41 and 7.35% (*P* < 0.01), the corrected incidence was 1/862 and 1/2560 (*P* < 0.05) in the suburban or rural area and urban area, respectively (**Table 1**).

Nine types of IEMs were diagnosed in 41 cases. The incidences of amino acid disorders, fatty acid disorders, and organic acid disorders were 1/2841, 1/5366, and 1/3019, respectively. Methylmalonic acidemia, phenylketonuria, and primary carnitine deficiency ranked the top 3 of all detected IEMs with the incidence of 1/3220, 1/4391, and 1/9659, respectively. The incidences of maple syrup urine disease, argininemia, ornithine transcarbamylase deficiency, short-chain acyl-CoA dehydrogenase deficiency, glutaric acidemia type II, and glutaric acidemia type I were 1/24,149, 1/16,099, 1/48,297, 1/12,074, 1/48,297, and 1/48,297, respectively.

### Genetic Analysis in the Confirmed Cases and Carriers

As IEMs are mainly caused by IEMs-associated mutations, genetic analysis is a golden approach to figure out the etiology of IEMs. Among the 41 confirmed cases, the parents of 28 cases were consent to perform genetic analysis. Thirty mutations in nine genes were detected, which resulted in seven types of IEMs (**Table 2**). Pathogenic mutations in the *PAH* and *PTS* genes were detected in four and one phenylketonuria cases, respectively. Pathogenic mutations in the *MMACHC* and *MUT* genes were detected in 11 and 2 cases of methylmalonic acidemia, respectively. Moreover, four cases of primary carnitine deficiency had mutations in the *SLC22A5* gene while no genetic mutation was detected in one primary carnitine deficiency case. In addition, mutations in *ACADS* gene were detected in four cases of short-chain acyl-CoA dehydrogenase deficiency, and two of them *were* accompanied with glutaric acidemia (type I or type II ) carried mutations in the *GCDH* and *ETFDH* genes, respectively. Interestingly, most cases were heterozygous except that two cases were homozygous, case 12 with short-chain acyl-CoA dehydrogenase deficiency and case 25 with methylmalonic acidemia (**Table 2**).

**Table 2:** Spectrum of variants in confirmed cases.

IEM	Gene Accession No.	Variant	Case
Phenylketonuria	*PAH* NG_008690	c. 728G>A (p. R243Q)/ c. 1315+6T>A (splicing error) c. 611A>G (p. Y204C)/ c. 721C>T (p. R241C) c. 1252A>C (p. T418P)/ c. 971T>A (p. I324N) c. 1139C>T (T380M)/ c. 1197A>T (V399V)	1 2 3 4
	*PTS* NG_008743	c. 166G>A (p. V56M)/ c. 286G>A (p. D56N)	5

Ornithine transcarbamylase deficiency	*OTC* NG_008471	c. A916G (p. R306G)	6

Primary carnitine deficiency	*SLC22A5* NG_008982	c. 1400C>G (p. S467C) c. 1400C>G (p. S467C) c. 1400C>G (p. S467C)/ c. 428C>T (p. P143L) c. 680G>A (R227H)	7 8 9 10
	/		11

Short-chain acyl-CoA dehydrogenase deficiency	*ACADS* NG_007991	c. 164C>T (p. P55L)^#^ c. 1031A>G (p. E344G)	12 13

Short-chain acyl-CoA dehydrogenase deficiency/glutaric acidemia type II	*ACADS/ETFDH* NG_007991/NG_007078	c. 164C>T (p. P55L)/ c. 770A>G (p. Y257C)	14

Short-chain acyl-CoA dehydrogenase deficiency/glutaric acidemia type I	*ACADS/GCDH* NG_007991/NG_009292	c. 164C>T (p. P55L)/c. 1064G>A (p. R355H)	15

Methylmalonic acidemia	*MMACHC* NG_013378	c. 567dupT (p. I190Yfs^∗^13) c. 445_446insA (p. C149^∗^) /c. 609G>A (p. W203^∗^) c. 217C>T (p. R73^∗^)/ c. 609G>A (p. W203^∗^) c. 1A>G (p. M1V) c. 482G>A (p. R161Q)/ c. 609G>A (p. W203^∗^) c. 567dupT (p. I190Yfs^∗^13) c. 609G>A (p. W203^∗^)/ c. 626dupT (p. T210Dfs^∗^35) c. 394C>T (p. R132^∗^) c. 658delAAG (p. K220del) c. 315C>G (p. Y105^∗^)/ c. 609G>A (p. W203^∗^) c. 609G>A (p. W203^∗^)^#^ c. 482G>A (p. R161Q)/ c. 567dupT (p. I190Yfs^∗^13)	16 17 18 19 20 21 22 23 24 25 26
	*MUT* NG_007100	c. 323G>A (R108H)/ c. 494A>G (D165G) c. 1106G>A (R369H)	27 28

*The symbol “#” in superscript represents homozygous and “/” represents no mutation.*

## Discussion

A total of 48,297 neonates were recruited for expanded newborn screening by MS/MS and the corrected incidence of IEMs is 1/1110, which was higher than the average incidences of IEMs in China (1/3795) (Shi et al., 2012) and in many other countries, such as 1/2500 in Canada (Applegarth et al., 2000), 1/2900 in Germany (Lindner et al., 2011), 1/1944 in Egypt (Hassan et al., 2016), 1/2916 in Malaysia (Yunus et al., 2016), 1/2800 in South Korea (Yoon et al., 2005), and 1/3165 in Singapore (Lim et al., 2014). Moreover, the incidence of IEMs in Jining was higher than that in many areas or cities of China, e.g., 1/1683 in Zibo, 1/4384 in Yancheng, 1/5626 in Zhejiang province, and 1/8304 in Nanning (Huang et al., 2011; Fan et al., 2013; Zhu et al., 2015; Lu et al., 2016). It indicated that the incidence of IEMs was greater in Jining area than in many regions.

It is known that the genetic variants related to IEMs are mainly detected by Sanger sequencing and MassARRAY (Wright et al., 2008; Zhu et al., 2010; Shibbani et al., 2014; Mutlu-Albayrak et al., 2015). Recently, next generation sequencing (NGS) started to be applied for the clinical genetic analysis of IEMs (Qian et al., 2017; Smon et al., 2018). Sanger sequencing is the golden standard to determine the DNA sequence, which is applied for the known gene locus in genetic analysis. Both MassARRAY and NGS are high throughput sequencing methods, which can be used for genetic analysis of a spectrum of genes. Compared with NGS, MassARRAY has been used more widely and longer to detect known mutations. In addition, it is reliable and the cost is less. Therefore, 942 sites in IEMs-associated genes were detected by MassARRAY in this study. However, NGS has the advantage to detect the novel mutations. Thus, it may be a good option for the case 11 to discover unknown mutations.

Although the corrected incidence was significantly higher in the suburban and rural area compared with that in the urban area, 1/862 vs. 1/2560, it is unlikely that the high incidence of IEMs in the suburban and rural area is caused by consanguineous marriage and close genetic relationship. First, consanguineous marriage including cousin marriage is prohibited by Chinese Marital Law, which is widely accepted and strictly abided by the residence. Moreover, genetic analysis showed that only one case was homozygous in 22 cases in the suburban area, while one in six cases was homozygous in the urban area. The aforementioned evidence indicated that the close genetic relationship is not the cause of high incidence of IEMs in the suburban/rural area. However, the underlying reasons of the difference need to be further investigated, such as environmental factors and life style.

The incidence of phenylketonuria varies in countries and regions (Williams et al., 2008). For example, the incidence of phenylketonuria varies from 1/7325 to 1/39,338 in countries of southeastern Europe, while the incidences are 1/11,400 in the United States and 1/4172 in Turkey (Hertzberg et al., 2011; Zerjav Tansek et al., 2015). The incidence of phenylketonuria is 1/11,614 in China based on the newborn screening data of past 30 years (Shi et al., 2012). However, we found that the incidence of phenylketonuria was 1/4391 in Jining, which was much higher than the incidence of phenylketonuria in China. Genetic analysis showed that six cases carried *PAH* mutants and one case carried *PTS* mutant in patients with phenylketonuria, suggesting that mutations in *PAH* gene are the predominant cause of phenylketonuria in Jining.

The incidence of methylmalonic acidemia was 1/3220 in Jining, which was close to 1/3920 in Jinan, a nearby city (Han et al., 2016). However, it was significantly higher than that in other cities of China, e.g., 1/26,000 in Shanghai/Beijing, and that in other countries, e.g., approximately 1/15,155 in an Italy population and 1/67,376 in Japan (Yamaguchi, 2008; Tu, 2011; Scolamiero et al., 2015). Genetic analysis was performed in 13 confirmed cases, among which 11 cases carried mutations in the *MMACHC* gene, suggesting that mutations in the *MMACHC* gene are the predominant cause of methylmalonic acidemia in Jining.

The incidence of primary carnitine deficiency was 1/9659, which was higher than that in other regions of China, e.g., 1/32,354 in Zhejiang province and 1/31,773 in Guangxi province (Huang et al., 2011). It was also higher than that in other countries, e.g., approximately 1/40,000 in Japan and 1/50,000 in the United States (Koizumi et al., 1999; Magoulas and El-Hattab, 2012). Among the five primary carnitine deficiency cases, four cases carried *SLC22A5* mutants. It suggested that *SLC22A5* mutants are the predominant cause of primary carnitine deficiency in Jining. However, no known mutation was detected in *SLC22A5* gene in case 11. It has to be noted that only the known mutations in the exons of *SLC22A5* gene can be detected by MassARRAY method in this study. Thus, novel mutations in the exons of *SLC22A5* gene may not be detected by this method. In addition, it is possible that mutations in exons of other genes may cause primary carnitine deficiency. Therefore, whole exon sequencing may need to be performed to discover the novel mutations in the exons of *SLC22A5* gene and/or other genes. In addition to mutations in the exons, mutations existing in the 5′-UTR, introns and 3′-UTR may contribute to the dysfunction of SLC22A by altering the expression level or splicing process. For example, an SNP in the intron 4 of *TMP21* gene significantly affects the splicing efficiency leading to the dysregulation of TMP21 expression, contributing to the pathogenesis of Alzheimer’s disease (Zhang et al., 2018). Therefore, the 5′-UTR, introns and 3′-UTR may also need to be sequenced in the later study.

In the present study, we showed that the incidence of IEMs was 1/1178 in Jining, while the incidence was significantly higher in the suburban and rural area compared with that in the urban area, 1/862 vs. 1/2560. The incidence of methylmalonic acidemia, phenylketonuria, and primary carnitine deficiency ranked the top 3 of all detected IEMs. In addition, 30 mutations in nine IEMs-associated genes were identified in the 28 confirmed cases and 19 cases with the mutations in *PAH*, *SLC22A5*, and *MMACHC* genes, respectively. Our work indicated that the overall incidence of IEMs is higher in Jining than that in China. Methylmalonic acidemia, phenylketonuria, and primary carnitine deficiency were the major IEMs and the mutations in *PAH*, *SLC22A5*, and *MMACHC* genes are the leading causes of IEMs resulting in phenylketonuria, primary carnitine deficiency, and methylmalonic acidemia, respectively, in Jining area. Therefore, it is critical to increase the coverage of expanded newborn screening by MS/MS and prenatal genetic consulting in Jining area. Furthermore, the underlying reasons of higher incidence of IEMs in Jining and particularly higher incidence of IEMs in the suburban area need to be investigated to achieve the decrease of the incidence of IEMs in the future.

## Author Contributions

KG and XC performed screening by MS/MS and collected the data. YW and XZ formulated the study, analyzed the data, wrote the manuscript, and designed the tables. CL and QK provided intellectual thoughts, revised the manuscript, and led the project.

## Conflict of Interest Statement

The authors declare that the research was conducted in the absence of any commercial or financial relationships that could be construed as a potential conflict of interest. The reviewer RTA and handling Editor declared their shared affiliation.
